# Protease, Growth Factor, and Heparanase-Mediated Syndecan-1 Shedding Leads to Enhanced HSV-1 Egress

**DOI:** 10.3390/v13091748

**Published:** 2021-09-01

**Authors:** Ghadah A. Karasneh, Divya Kapoor, Navya Bellamkonda, Chandrashekhar D. Patil, Deepak Shukla

**Affiliations:** 1 Department of Microbiology and Immunology, College of Medicine, University of Illinois at Chicago, 835 S. Wolcott, Chicago, IL 60612, USA; ghadah.karasneh@uky.edu (G.A.K.); dkapoo5@uic.edu (D.K.); 2 Department of Ophthalmology and Visual Sciences, College of Medicine, University of Illinois at Chicago, 1855 W. Taylor, Chicago, IL 60612, USA; navyab3@uic.edu (N.B.); cdpatil@uic.edu (C.D.P.)

**Keywords:** heparan sulfate, heparan sulfate proteoglycan, HSV-1

## Abstract

Heparan sulfate (HS) and heparan sulfate proteoglycans (HSPGs) are considered important for the entry of many different viruses. Previously, we demonstrated that heparanase (HPSE), the host enzyme responsible for cleaving HS chains, is upregulated by herpes simplex virus-1 (HSV-1) infection. Higher levels of HPSE accelerate HS removal from the cell surface, facilitating viral release from infected cells. Here, we study the effects of overexpressing HPSE on viral entry, cell-to-cell fusion, plaque formation, and viral egress. We provide new information that higher levels of HPSE reduce syncytial plaque formation while promoting egress and extracellular release of the virions. We also found that transiently enhanced expression of HPSE did not affect HSV-1 entry into host cells or HSV-1-induced cell-to-cell fusion, suggesting that HPSE activation is tightly regulated and facilitates extracellular release of the maturing virions. We demonstrate that an HSPG-shedding agonist, PMA; a protease, thrombin; and a growth factor, EGF as well as bacterially produced recombinant heparinases resulted in enhanced HSV-1 release from HeLa and human corneal epithelial (HCE) cells. Our findings here underscore the significance of syndecan-1 functions in the HSV-1 lifecycle, provide evidence that the shedding of syndecan-1 ectodomain is another way HPSE works to facilitate HSV-1 release, and add new evidence on the significance of various HSPG shedding agonists in HSV-1 release from infected cells.

## 1. Introduction

Herpes simplex virus type-1(HSV-1) is a member of the alpha herpesvirus subfamily of herpesviruses. It is a neurotropic virus capable of infecting the nervous system and causing neurological diseases. HSV-1 establishes latency in the host sensory neurons and thereafter reactivates sporadically to cause human sufferings [1]. HSV-1 infection can be mostly asymptomatic, but it can also result in serious diseases including blindness, meningitis, and encephalitis. Fever blisters are the most common attributes of an HSV-1 disease. It is a leading cause of infectious corneal blindness in the developed countries, and sporadic, fatal encephalitis worldwide [2,3]. Current treatment of HSV-1 infection is acyclovir or similar nucleoside analogs that target virus replication [4]. However, there is no effective treatment to eradicate the virus from the body or vaccination against HSV-1. Moreover, the therapeutic effectiveness is frequently compromised by the emergence of drug-resistant virus isolates especially in immunocompromised individuals [5]. Therefore, understanding the molecular basis of viral entry and spread will help in the development of new strategies to prevent the infection.

HSV has low species specificity and a wide host range, and it is capable of infecting most cell types in vitro [6]. HSV-1 entry into host cells is a multistep process that starts with HSV-1 attachment through its glycoproteins gB and gC to heparan sulfate proteoglycans (HSPGs) on the cell surface [7,8]. Although each glycoprotein plays a specific role in viral entry and attachment, the role of gC as an essential or non-essential protein has always been debatable and thus of prime interest, too. Its roles in virus entry and its potential as an antigenic determinant are still widely explored [9]. Generally, in the process of HSV-1 fusing with the plasma membrane and entering the cell, glycoprotein D (gD) must bind to the herpesvirus entry mediator (HVEM). Heparan sulfate (HS) is a glycosaminoglycan (GAG) attached to the protein core of HPSGs expressed on the cell surface [10]. Its presence is important for HSV-1 to efficiently enter into cells [11]. Syndecans are transmembrane proteoglycans found in the extracellular membrane that contain attachment sites for HS chains [12]. The four types of syndecans found in mammal cells possess different domains, which include an N-terminal signal peptide (ectodomain) and a C-terminal cytoplasmic domain [13]. As a proteoglycan responsible for much of the presence of HS near the cell surface, syndecan expression has an inherent potential to prevent viral egress by trapping the exiting virions. The regulation of syndecan-1 shedding is a common mechanism invoked by HSV-1 to promote the exit of new infectious virions from the cell [14,15,16,17]. The shedding of syndecan-1 is a natural process and is achieved through the proteolytic cleavage of the cell-surface ectodomains. Agents that serve to accelerate the syndecan-1 shedding process would lead to an increase in viral egress.

PMA, thrombin, and epidermal growth factor (EGF) are shedding agonists implicated in the acceleration of syndecan-1 shedding [18]. Thrombin and EGF are compounds commonly associated with wound repair and are known to accelerate shedding through the activation of G-protein coupled receptors (GPCR) and receptor tyrosine kinases (RTK), respectively [18]. HPSE is an endo-β-D-glucuronidase that has the ability to cleave HS side chains [19,20]. The cleavage of HS results in the disassembly of the extracellular matrix (ECM) and basement membrane underlying epithelial and endothelial cells [21,22]. HPSE has functions that are related to its enzymatic activity including tumor metastasis, angiogenesis, embryo implantation, wound healing, inflammation, and autoimmunity [23,24,25]. Syndecan-1 shedding also requires HPSE activity and it is considered a shedding agonist [26,27].

This study demonstrates that HPSE overexpression resulted in a significant increase in virus egress. We show that HPSE facilitates HSV-1 egress not only by degrading HS chains but also by inducing syndecan-1 shedding. Moreover, treating HSV-1 infected cells with the syndecan shedding agonists also results in an enhanced virus release from infected cells. Collectively, we show that shedding agonists indiscriminately facilitate HSV-1 release from infected cells. 

## 2. Materials and Methods

### 2.1. Cell Culture and Viruses

Wild-type Chinese hamster ovarian (CHO-K1) cells, African green monkey kidney cells (Vero) cells, and gL-expressing Vero cells (79B4) were provided by P. G. Spear (Northwestern University). Human cervical (HeLa) cells were provided by B. S. Prabhakar (University of Illinois at Chicago, Chicago, IL, USA). The human corneal epithelial (HCE) cell line (RCB1834 HCE-T) was provided by Kozaburo Hayashi (National Eye Institute, Bethesda, MD, USA) [28]. The authenticity of the cell lines was ensured by STR profiling. The CHO cell line was grown in Ham’s F-12 medium (Gibco/BRL, Carlsbad, CA, USA) supplemented with 10% fetal bovine serum (FBS) and penicillin and streptomycin (*p*/S) (Sigma-Aldrich, St. Louis, MO, USA). Vero, 79B4, and HeLa cells were grown in Dulbecco’s modified Eagle’s medium (DMEM) supplemented with 10% FBS and *p*/S (Sigma). HCE cells were grown in minimum essential medium (MEM) supplemented with 10% FBS and *p*/S. Wild-type HSV-1 (KOS) virus strain, β-galactosidase expressing recombinant HSV-1 (KOS) gL86, and syncytial plaque-forming HSV-1 (KOS) 804 virus were provided by P.G. Spear (Northwestern University). Virus stocks were propagated and titered on Vero cells and stored at −80 °C. 

### 2.2. Plasmids, Antibodies and Reagents

The HSV-1 (KOS) glycoproteins-expressing plasmids used were pPEP98 (gB), pPEP99 (gD), pPEP100 (gH), and pPEP101 (gL) [29]. Plasmids used for Luciferase reporter assay were pCAGT7 (T7 RNA polymerase) and pT7EMCLuc, which were described previously [29]. Human heparanase expression plasmid pIRES2 EGFP-HPSE1 (HPSE) and the control empty vector pIRES2 EGFP were provided by Dr. Ralph Sanderson (University of Alabama at Birmingham, Brimingham, AL, USA) [26]. The following antibodies were used in this study: rabbit polyclonal anti-heparanase (sc-25825, Santa Cruz Biotechnology, Santa Cruz, CA, USA; 1:200–1:1000), rabbit GAPDH mAb (G-9545, Sigma-Aldrich, St. Louis, MO, USA; 1:2000), anti-human syndecan-1 antibody (sc-12765, Santa Cruz Biotechnology, Santa Cruz, CA, USA; 0.6 µg/mL), horseradish peroxidase-conjugated anti-rabbit IgG (73102, Jackson ImmunoResearch Laboratories, West Grove, PA, USA; 1:20,000), and horseradish peroxidase-conjugated anti-mouse IgG (115-035-062, Jackson ImmunoResearch Laboratories, West Grove, PA, USA; 1:250,000). Heparinase II and III enzymes were provided by Dr. Jian Liu (University of North Carolina, Chapel Hill, NC, USA) and used at either 15 or 30 IU/mL PMA (4α-phorbol-12,13 didecanoate) (P1585, Sigma-Aldrich, St. Louis, MO, USA).

### 2.3. HSV-1 Entry Assay

Standard entry assays were used as described previously [16]. Cells were grown in 96-well plates until subconfluency. After 16 h, cells were transfected with heparanase expression plasmids using Lipofectamine 2000 (Life Technologies Incorporation, Eugene, OR, USA). Media was changed 6 h post transfection. Then, 24 h post transfection, cells were infected in a two-fold serial dilution with the recombinant HSV-1 virus expressing β-galactosidase enzyme HSV-1 (gL86). Cells were incubated with virus for 6 h at 37 °C. Then, cells were washed twice with PBS-ABC, and the soluble substrate for the β-galactosidase enzyme o-nitrophenyl-β-d-galactopyranoside (ONPG) was added. Enzymatic activity was measured at 410 nm using an ELISA plate reader (Spectra Max 190, Molecular Devices, Sunnyvale, CA, USA).

### 2.4. Cell-to-Cell Fusion Assay

Standard cell-to-cell fusion assay was used as previously described [13]. Cells were split into two populations. “Target” cells were transfected with plasmid expressing Nectin-1 as a gD receptor (1.0 µg) and the luciferase gene (0.5 µg). “Effector” cells were transfected with plasmids expressing HSV-1 glycoproteins gD, gB, gH, and gL and T7 RNA polymerase (0.5 µg each). After 16 h, target and effector cells were mixed in a 1:1 ratio and replated in 24-well dishes. Luciferase activity was measured after 16 h. As a negative control, target cells were mixed with effector cells that lack HSV-1 gB. In order to examine the role of heparanase during cell-to-cell fusion, target cells were additionally transfected with 0.5 µg of a plasmid expressing human heparanase (HPSE) or control empty vector. 

### 2.5. Immunoblotting

For experiments after HPSE overexpression, cells were lysed 24–48 h post transfection using cell lysis buffer supplemented with protease inhibitor. For immunoblotting experiments after HSV-1 infection, cells were infected with HSV-1 at a multiplicity of infection (MOI) of 1 for 2 h at 37 °C. Then, cells were washed with PBS 3×, complete media was added, and incubation was continued. At 24 h post infection, cells were lysed with cell lysis buffer supplemented with protease inhibitor. Western blot assay was performed according to protocols described previously [13]. Briefly, whole cell lysates were denatured in NuPAGE LDS Sample Buffer (NP0007, Invitrogen, Grand Island, NY, USA) and heated to 86 °C for 8 min before gel loading. Equal amounts of protein were subjected to 4–12% SDS-PAGE and electroblotted onto a nitrocellulose membrane. Nonspecific binding was blocked using 5% nonfat milk in tris-buffered saline (TBS) for 2 h at 37 °C. Then, the membranes were incubated with primary rabbit polyclonal antibodies against heparanase overnight at 4 °C. The blots were rinsed 5 times with 0.1% TTBS (0.1% Tween 20 in TBS) for 5 min followed by incubation for 1 h at room temperature with horseradish peroxidase-conjugated anti-rabbit IgG. Protein bands were detected using a SuperSignal West Femto maximum sensitivity substrate (Pierce, 34096) and visualized using an ImageQuant LAS 4000 imager (GE Healthcare Life Sciences). Protein bands were quantified using ImageQuant TL image analysis software (version: 7). GAPDH was measured as a loading control. The densitometry quantification of the blot was performed using ImageQuant TL image analysis software (GE Healthcare Life Sciences).

### 2.6. Cytotoxicity Assays

Cytotoxicity assays were performed after heparanase overexpression. Approximately 1.5 × 10^4^–2.5 × 10^4^ cells were plated in a 96-well plate. Cells were transfected with heparanase expression plasmid or control empty vector. After 6 h, complete media (supplemented with FBS and *p*/S) was added to cells. Then, 24 h post transfection, growth medium was removed, and 100 uL of complete media was added to each well in addition to 20 µL of Cell Titer 96 Aqueous One solution (Promega, Madison, WI, USA). Plates were incubated at 37 °C for 1–4 h. Then, absorbance was measured at 490 nm using a microplate ELISA reader (Spectra Max 190 Molecular Devices, Sunnydale, CA, USA). Background absorbance was subtracting from the reading, which represents cell-free wells filled only with media and the Cell Titer 96 Aqueous One solution.

### 2.7. Plaque Assays

Monolayers of cells in 6-well plates overexpressing heparanase or control empty vector (0.75 µg using Lipofectamine 2000 reagent) were infected with 10-fold serial dilutions of syncytial plaque-forming HSV-1 (KOS) 804 virus stocks. Infected cells were fixed with 100% methanol for 5 min, stained with crystal violet, and a collective size of 20 or approximate plaques were measured in pixels on GraphPad Prism software, and only the strain-specific plaques were counted [30].

### 2.8. HSV-1 Egress Assay after HPSE Overexpression

HeLa cells grown in 6-well culture plates were transfected with heparanase expression plasmid pIRES2 EGFP-HPSE1 (HPSE) or the control empty vector pIRES2 EGFP. After 18 h, cells were infected with HSV-1 (KOS) at an MOI of 0.1 for 2 h at 37 °C. Then, cells were washed 3× with Hanks’ balanced salt solution, and growth medium was added back to the cells, and incubation was continued at 37 °C. At 6, 12, 24, 36, 48, and 60 h post infection, 100 µL of cell culture supernatant were collected and titered using Vero cells monolayers in 24-well plates. Each time the supernatant was collected, 100 µL complete medium was added to maintain the total volume of growth media in the wells. 

### 2.9. HSV-1 Release after Heparinase Treatment

HeLa and HCE cells grown in 6-well culture plates were infected with HSV-1 (KOS) at an MOI of 0.1 and 0.01 respectively for 2 h at 37 °C. Then, cells were washed 3× with Hanks’ balanced salt solution and then treated for 1 min with citrate buffer (PH = 3.0) and washed. Afterwards, cells were treated with either heparinase III (15 and 30 IU/mL) or a combination of heparinases II and III (15 and 30 IU/mL) for 2 h at 37 °C. After 2 h, cells were washed, growth medium was added back to the cells, and incubation was continued for 20 h at 37 °C. HSV-1 release was measured by titering the supernatant of the cultured cells on Vero cells. As a control, some cells were infected with HSV-1 (KOS) but were not treated with the heparinases.

### 2.10. HSV-1 Release after PMA, Thrombin, and EGF Treatment

HeLa and HCE cells were infected at an MOI of 0.01 with HSV-1 (KOS) for 2 h. After 2 h, cells were washed 3× with Hank’s balanced salt solution, and cells were overlaid with complete medium, and incubation was continued for 8 h at 37 °C. Then, cells were washed 3× with serum-free medium and were serum starved for 12 h. After 12 h, cells were treated with PMA, thrombin, and EGF in serum-free medium for 0.5, 1, 2, and 4 h. After each time point, 100 µL of the cell culture supernatant was titered on Vero cells monolayer. HSV-1-infected cells treated with DMSO were used as control.

### 2.11. Immunofluorescence Microscopy

HeLa cells and HCE cells were cultured in glass-bottom dishes (MatTek Corporation, Ashland, MA, USA). At 40% of confluency, the cells were serum starved for 12 h. Then, cells were treated with DMSO, PMA, thrombin, and EGF for 4 h. After 4 h post-treatment, cells were fixed in 10% paraformaldehyde for 15 min. The cells were washed with PBS, which was followed by incubation with PBS with 1% bovine serum albumin (BSA) at 4 °C for 1 h. Then, the cells were washed with PBS buffer and incubated with the primary antibody at a dilution of 1:100 for 1 h at 4 °C. The primary antibody was removed by washing with PBS again, and cells were incubated with FITC-conjugated secondary antibody for heparan sulfate (green) and Alexa fluor 647 (red) for syndecan-1 expression on the cell surface at a dilution of 1:100 for 1 h and were examined under the Zeiss 710 confocal microscope (Jena, Germany) using a ×63 oil immersion objective lens with the pinhole set to 1 AU.

### 2.12. Flow Cytometry

Cells’ surface expression of syndecan-1 was detected after infection with HSV-1 KOS-WT. HCE and HeLa cells were grown in 12-well plates until confluency. Then, cells were infected with HSV-1 (KOS) at an MOI of 0.1 for 2 h before being changed into cultured media for 8 h. Then, cells were incubated with serum-free media for 12 h followed by DMSO, thrombin, PMA, or EGF treatment. Cells were collected after 4 h and incubated for 1 h with 3 μg of anti-human syndecan-1 antibody in each sample. All the cells were washed with FACS buffer and then incubated with anti-mouse syndecan-1 antibody (Alexa Fluor 647, Billerica, MA, USA) at 4 °C for 1 h. Then, the cells were washed with FACS buffer and run on the flow cytometer (CytoflexS flow cytometer). Basal cell surface expression of both syndecan-1 and HS was detected after growing HeLa and HCE cells in 6-well plates until confluency. Cells were collected, washed with FACS buffer, and stained with the same antibodies and run on the flow cytometer.

### 2.13. Slot Blot Assay

A previously described protocol was followed with some modifications [31]. Serum-starved HeLa and HCE cells were treated with PMA, DMSO, thrombin, or EGF in serum free media. Additionally, HeLa and HCE cells were infected and treated as described in the flow cytometry procedure. Then, 100 μL supernatant from each of 12 wells (96-well plate) or 300 μL supernatant from 1 well (24-well plate) were added to 1 mL of acidification buffer (Buffer A) (150 mM NaCl, 50 mM NaOAc, 0.1% Tween-20, pH 4.5). Immobilon NY+ membrane (INYC00010, Millipore Corporation, Billerica, MA, USA) was prepared by first soaking it in buffer A. By acidifying the samples in buffer A, only highly anionic molecules in the supernatant, including proteoglycans, are retained by the cationic Immobilon-NY+ membrane. Most proteins are cationic at this pH and pass through the membrane. A Bio-Dot SF Apparatus (170-6542, Bio-Rad, Hercules, CA, USA) was used for syndecan-1 slot blot under mild vacuum. The sample wells were re-saturated with 100 μL of buffer A; then, 400 μL of sample solution was used per well on the apparatus. Samples were kept in the wells on top of the membrane for 3 min, and then, it was drawn through the membrane for another 3 min. Then, the wells were rinsed twice with 400 μL of buffer A. This was repeated with a second membrane and the infected samples. The membranes were taken out of the apparatus, rinsed twice for 5 min in buffer A, and blocked with 3% milk in buffer B (150 mM NaCl, 10 mM Tris-HCl, pH 7.4) for 1 h. Then, one membrane was incubated with anti-human syndecan-1 antibody (sc-12765, Santa Cruz Biotechnology, Santa Cruz, CA, USA; 1:500) in 3% milk in buffer C (150 mM NaCl, 10 mM Tris-HCl, 0.3% Tween-20, pH 7.4) overnight on a shaker at 4 °C. The other membrane was incubated with Anti-HSV1 + HSV2 gD antibody (ab6507, Abcam, Cambridge, UK; 1:1000) in 3% milk in buffer C (150 mM NaCl, 10 mM Tris-HCl, 0.3% Tween-20, pH 7.4) for 1 h on a shaker at room temperature. Then, the membranes were washed with buffer C 3× for 15 min and incubated with horseradish peroxidase-conjugated anti-mouse IgG (115-035-062, Jackson ImmunoResearch Laboratories, West Grove, PA, USA; 1:2000). Then, membranes were developed using the SuperSignal West Femto maximum sensitivity substrate (Thermo Scientific, Waltham, MA, USA) with an ImageQuant LAS 4000 biomolecular image (GE Healthcare Life Sciences, Pittsburgh, PA, USA). Densitometry quantification of the slot blot was performed using ImageQuant TL image analysis software (GE Healthcare Life Sciences).

### 2.14. MTT Assay

The MTT assay was performed as an indicator of cell viability using various concentrations of PMA, thrombin, and EGF on HeLa and HCE cells plated at a density of 2 × 10^4^ per well in 96-well plates overnight. Concentrations starting at 5 µM for PMA, 50 ng/mL for thrombin, and 25 mg/mL for EGF were two-fold serially diluted in complete media and overlayed on cell monolayers for 4 h. After incubation, 3-(4,5-dimethylthiazol-2-yl)-2,5-diphenyltetrazolium bromide (MTT; BioVision, Milpitas, CA, USA) was added to cells at a concentration of 0.5 mg/mL in whole media and incubated for 3 h to allow crystal formation. The formazan crystals were dissolved by adding acidified isopropanol (1% hydrochloric acid *v*/*v*) on the cells and incubated for 1 h. Dissolved violet crystals were transferred to new 96-well plates and analyzed by a micropate reader (TECAN GENious Pro) at 510 nm. 

### 2.15. Statistical Analyis

Statistical analysis was performed using GraphPad Prism software (version 9.0). A one-way ANOVA, Dunnett’s test was performed to evaluate the effect of heparinases treatment on HSV-1 release from infected cells, comparing with the DMSO control. A two-way ANOVA, Bonferroni test was performed to evaluate the effect of PMA, thrombin, and EGF treatment on viral egress relative to DMSO. A paired, two-tailed, Student’s *t* test was used to analyze the rest of the data. Asterisks denote a significant difference as determined by Student’s *t*-test: *, *p* < 0.05; **, *p* < 0.01; ***, *p* < 0.001; ****, *p* < 0.0001.

## 3. Results

### 3.1. HPSE Overexpression Does Not Affect Cell Viability, Cell Entry, and Cell–Cell Fusion in HeLa and HCE Cells

**Figure 1:**
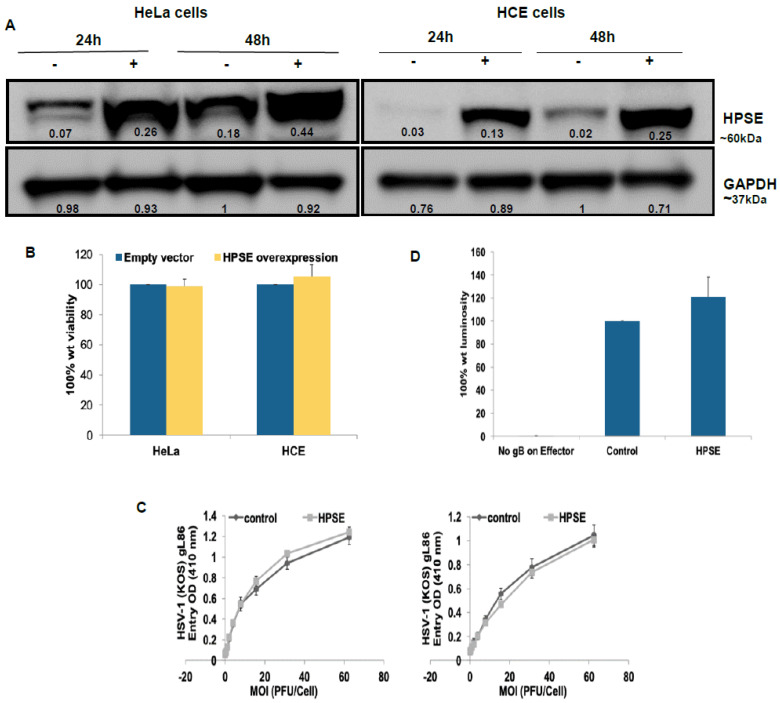
HPSE overexpression does not affect cell viability, cell entry, and cell–cell fusion in HeLa and HCE cells. (**A**) HeLa and HCE cells were transfected with human HPSE expression plasmid or with a control empty vector (control). Then, 24 and 48 h post transfection immunoblot of cell lysates were prepared and probed with anti-HPSE antibody. −: un-transfected cells, +: transfected cells. GAPDH protein level was measured as loading control. Representative blots of three independent experiments are shown. The densitometry quantification values are represented on the blot indicating the signal intensity with respect to the GAPDH (control). (**B**) Cells were grown in 96-well plates, transfected with HPSE expression plasmid or with a control empty vector. 24 h post transfection, triplicate wells were evaluated for cell viability using MTS assay. Results are expressed as 100% wild-type (wt) viability where they represent the percent corrected absorbance after subtracting the background absorbance, relative to empty vector transfected cells, and are mean ± 1 SD of three independent experiments. (**C**) HeLa and HCE cells were transfected with either HPSE expression plasmid (HPSE) or an empty vector (control). 24 h post transfection, cells were infected in a twofold serial dilution with the recombinant HSV-1 virus expressing β-galactosidase enzyme HSV-1(gL86). Cells were incubated with virus for 6 h at 37 °C. Then, cells were washed, permeabilized, and incubated with ONPG substrate. Enzymatic activity was measured at 410 nm using an ELISA plate reader (Spectra Max 190, Molecular Devices, Sunnyvale, CA, USA). Results are presented as mean ± 1 SD of at least three independent experiments. (**D**) Target cells for CHO-K1 cells were either transfected with HPSE or with an empty vector (control) and mixed with effector cells 24 h post-transfection. As a negative control, target cells were mixed with effector cells that lack HSV-1 gB. Fusion was measured 16 h post mixing. Results are presented as mean ± 1 SD of five independent experiments.

In order to examine the importance of HPSE in the various aspects of HSV-1 infection, human HPSE expression plasmid pIRES2 EGFP-HPSE1 (HPSE) was used to induce the expression of HPSE in HeLa and HCE cells. The empty vector pIRES2 EGFP was used as a control. The overexpression of HPSE was confirmed at the protein level using Western blot analysis in both HeLa and HCE cells 24 and 48 h post transfection (Figure 1A). We confirmed the HPSE transfection by monitoring the expression of GFP, tagged to HPSE (Figure S1 in Supplementary Materials). The signal intensity of HPSE relative to GAPDH represented on the blot suggests the higher amount of HPSE being produced after transfection. The relative fold change in the signal intensity is 3.4 and 3.6 at 24 h post transfection and 2.3 and 9.8 at 48 h post-transfection in HeLa and HCE transfected cells with respect to untransfected cells, respectively. To evaluate the effect of enhancing HPSE expression on cell viability, MTT assay was performed 1 day post HPSE overexpression. Higher HPSE levels in HeLa and HCE cells showed no effect on cell viability (Figure 1B).

It is well established that HSV-1 utilizes HS on the surface of the host cell as an attachment receptor preceding virus entry into the host cell [15]. Moreover, HPSE has the important role of cleaving HS chains as part of the remodeling process of the extracellular matrix [32]. To investigate whether enhanced HPSE expression would affect HSV-1 ability to enter into the host cell, HPSE plasmid was again transfected into HeLa and HCE cells, and HSV-1 entry was evaluated. A previously described HSV-1 entry assay [17] was used to compare viral entry into cells with enhanced HPSE expression with those transfected with an empty vector. HeLa and HCE cells were infected with a recombinant β-galactosidase expressing HSV-1 (KOS) gL 86 reporter virus. The entry of HSV-1 was measured after 6 h of viral infection. As shown in Figure 1C, enhanced HPSE expression did not affect the ability of HSV-1 to enter into HeLa and HCE cells compared to control cells overexpressing an empty vector. HSV-1 induced cell-to-cell fusion is a process that resembles the viral fusion. It involves the fusion of plasma membrane of an infected cell with that of a neighboring uninfected cell as a way for viral spread [29]. Upon virus entry, viral glycoproteins are expressed on the surface of infected cells. This allows the binding and fusion of the viral glycoproteins on the surface of infected cells with neighboring uninfected cells that express the receptors for HSV-1 glycoproteins, forming large multinucleated cells called syncytia. The requirements for virus-induced cell-to-cell fusion are the same as those for virus entry: the glycoprotein gB, gD, gH, and gL, as well as a gD receptor [29]. Since HPSE overexpression showed no effect on HSV-1 entry, we sought to investigate the effect of enhancing HPSE expression on HSV-1 induced cell-to-cell fusion, which might reflect the scenario during virus membrane fusion during the initial infection.

To study HSV-1-induced cell-to-cell fusion following HPSE overexpression, a standard co-cultivating system was utilized. Two populations of cells, “Target” and “Effector”, were co-cultivated, which was followed by measuring the cell fusion between these two cell populations. Target cells express gD receptor and the luciferase reporter gene under the control of the T7 promoter. Effector cells express HSV-1 glycoproteins that are absolutely required for virus fusion (gB, gD, gH, and gL) plus T7 polymerase. Then, 16 h post mixing the two populations, cell-to-cell fusion was quantified by measuring the luciferase gene activity. As a negative control, target cells were mixed with effector cells that lack HSV-1 gB, which results in a dramatic reduction in HSV-1-induced cell-to-cell fusion. The CHO-K1 cell line that lacks the HSV-1 gD receptor was used. Enhancing HPSE expression on target cells resulted in a mild increase (20.8 in cell-to-cell fusion compared to control cells that overexpress an empty vector (*p* > 0.05) (Figure 1D). These results confirm that enhancing HPSE expression does not significantly affect virus entry or the virus-induced membrane fusion.

### 3.2. Transient Overexpression of HPSE Reduces HSV-1 Syncytial Spread and Instead Promotes Extracellular Release

**Figure 2:**
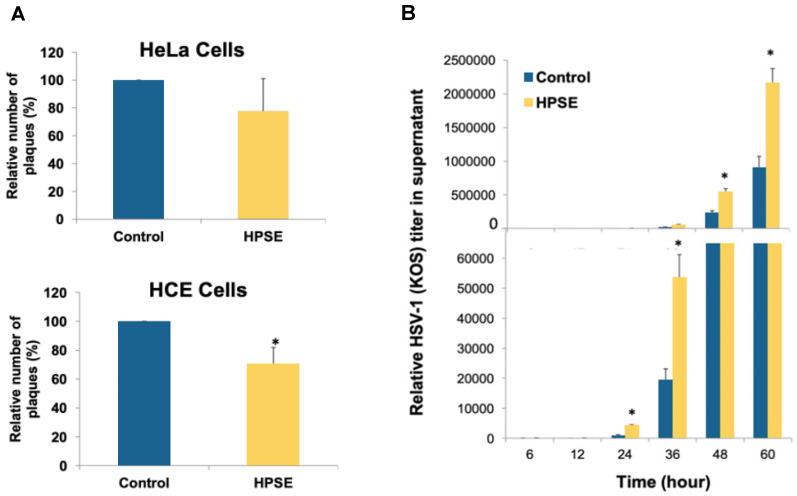
Transient overexpression of HPSE reduces HSV-1 syncytial spread and instead promotes extracellular release. (**A**) HeLa and HCE cells were transfected with either HPSE expression plasmid (HPSE) or with a control empty vector. Then, 24 h post transfection, cells were infected with syncytial plaque-forming HSV-1 (KOS) 804 virus at MOI of 0.001. Four to 5 days post infection, cells were fixed and stained with crystal violet stain. Infectivity was measured by the number of plaque-forming units (PFUs). Results are means ±1 SD of at least three independent experiments conducted in duplicate. (* *p* < 0.05). (**B**) HeLa cells were transfected with either HPSE expression plasmid (HPSE) or with a control empty vector. Then, 24 h post transfection, cells were infected with HSV-1 (KOS) for 2 h. After 2 h, cells were washed, and growth medium was added back to the cells. At time points: 6, 12, 24, 36, 48, and 60 h post infection, HSV-1 release from infected cells was examined by tittering 100 µL from the culture supernatant using plaque assay on Vero cells. A representative graph of two independent experiments conducted in duplicate is shown. (* *p* < 0.05).

Since overexpressing HPSE did not affect HSV-1 entry into HeLa and HCE cells, the effect of enhanced HPSE expression on virus plaque formation was analyzed [30]. Syncytial plaque formation reflects the ability of the virus to spread from cell to cell. Enhancing HPSE expression in HeLa and HCE cells reduced the plaque numbers by 22.5% (*p* > 0.05) and 29.6% (*p* ≤ 0.05) respectively compared to cells transfected with an empty vector (Figure 2A). This suggests that higher HPSE expression may tend to prevent syncytial spread of the virus. To determine whether it affects extracellular release of the virions, virus egress assay was performed in HeLa cells overexpressing HPSE plasmid. HeLa cells were either transfected with an empty vector or with HPSE expression plasmid. Then, 24 h post transfection, cells were infected with HSV-1 (KOS) for 2 h. Then, the cells were washed, growth media was added, and incubation was continued. At the indicated time points, 100 µL from the growth medium were collected and titered to measure virus release from the cells. Enhancing HPSE expression resulted in significantly higher HSV-1 release into the culture supernatant (*p* ≤ 0.05) (Figure 2B). These results verify our previous findings that HPSE plays an important role in extracellular virus egress, possibly through cleaving HS chains from infected cells.

### 3.3. Recombinant Heparinase Treatment of HSV-1 Infected HeLa and HCE Cells Induces HSV-1 Release from Infected Cells

**Figure 3:**
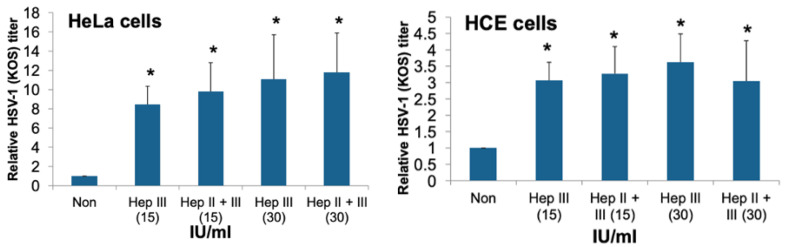
Recombinant heparinases treatment to HSV-1 infected HeLa and HCE cells induces HSV-1 release from infected cells. HeLa and HCE cells were infected at an MOI of 1 with HSV-1 (KOS) for 2 h. After 2 h, cells were treated with citrate buffer (pH = 3) for 1 min, which was followed by cell washing. Then, cells were either mock treated or treated with heparanase III or a combination of heparanase II and III, at 15 or 30 IU/mL for 2 h at 37 °C. After 2 h, cells were washed, and growth medium was added back to the cells. HSV-1 release from infected cells was examined after 20 h incubation at 37 °C post cell treatment by plaque assay using Vero cells. Results are means ± 1 SD of three independent experiments conducted in duplicate (* *p* < 0.05).

In order to confirm that HS removal from the cell surface is a mechanism for enhancing HSV-1 egress from infected cells, the effect of the addition of exogenous recombinant heparinases on HSV-1 egress was evaluated. HeLa and HCE cells were infected with HSV-1 (KOS) at MOI of 0.1 and 0.01 respectively for 2 h at 37 °C. Then, cells were washed, and bound virus was removed by citrate buffer (pH = 3) treatment, followed by cells washing. Then, cells were treated with either heparinase III (15 and 30 IU/mL), or a combination of both heparinases II and III (15 and 30 IU/mL) for 2 h at 37 °C. After 2 h, cells were washed, growth medium was added back to the cells, and incubation was continued for 20 h at 37 °C. HSV-1 release was measured by titering the supernatant of the cultured cells on Vero cells. As a control, some cells were infected but were not treated with the heparinases. Treating cells with heparinase III, or a combination of heparinase II and III at two different concentrations resulted in a significant induction in viral release from infected HeLa and HCE cells to the culture supernatant by 8–11 and 3–3.6 folds, respectively (*p* ≤ 0.05) (Figure 3). These results strengthen the notion that HPSE facilitates HSV-1 release.

### 3.4. PMA, Thrombin, and EGF Treatment Accelerates Syndecan-1 Shedding from HeLa and HCE Cells’ Surfaces to the Culture Supernatant

**Figure 4:**
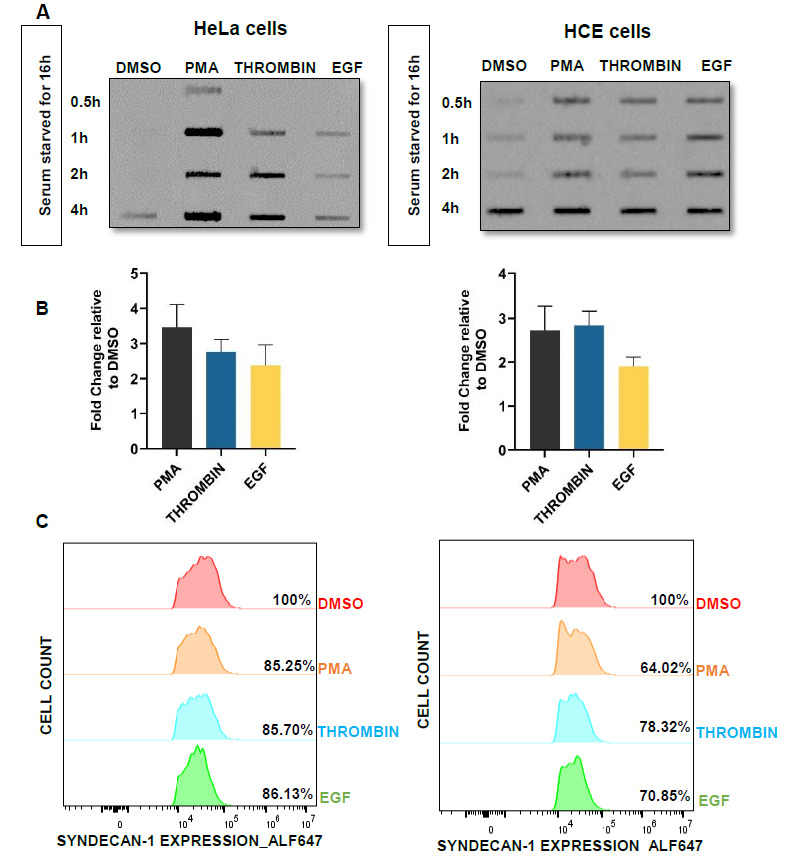
PMA, thrombin, and EGF treatment accelerates syndecan-1 shedding from HeLa and HCE cells’ surface to the culture supernatant. (**A**) HeLa and HCE cells were serum starved for 16 h. Then, cells were treated with DMSO, PMA, thrombin, or EGF in serum-free media. At 0.5, 1, 2, and 4 h time points, supernatant was collected. Shed syndecan-1 was evaluated by slot blot analysis. Results are representative of two independent experiments. Representative blots of two independent experiments are shown. (**B**) Densitometry quantification of the slot blot was performed using ImageQuant TL image analysis software. The results are shown as fold change of total syndecan-1 shedding after treatment with PMA, thrombin, and EGF in the supernatant over DMSO (control) cells. Representative graph of two independent experiments conducted in duplicate is shown. (**C**) Syndecan-1 expression in HeLa and HCE cells was measured using flow cytometry. Cells were infected HSV-1 (KOS) at an MOI of 0.1 for 2 h, and then, the media was changed into complete media for 8 h. Cells were further serum-starved for 12 h and then treated with DMSO (control), thrombin, PMA, or EGF for 4 h. After 4 h, cell lysates were collected and fixed with 4% PFA and then stained for syndecan-1. The results depict the decreased level of syndecan-1 expression on the surface of the cells treated with PMA, thrombin, and EGF than the DMSO (control)-treated cells.

Syndecan-1 is highly expressed on the epithelial cell surface but sheds off constitutively through different triggering factors that include proteases, chemokines, epidermal growth factors (EGF), and virulence factors. In order to look for factors that are thought to regulate syndecan-1 expression on the cell surface by accelerating its shedding to the culture supernatant, cells were treated with PMA (phorbol 12-myristate 13-acetate), which is a well-known shedding agonists of syndecan-1 [33], protease (Thrombin) and a growth factor (EGF), which are known to mediate the shedding by G-protein coupled receptor and protein tyrosine kinase regulated receptor, respectively, in the endothelial cells during wound repair. HeLa and HCE cells were initially serum starved for 16 h and then treated with PMA (1µM), thrombin (10 ng/mL), and EGF (5 mg/mL) in serum-free media. The MTT assay was performed to ensure the normal metabolic activity of the cells after treatment with PMA, thrombin, and EGF. The graph depicted that the percentage of cell viability relative to DMSO (control) at the applied concentration was approximately 75, 120 (PMA); 113, 90 (Thrombin); and 107, 94 (EGF) for HeLa and HCE cells, respectively (Figure S2). At 0.5, 1, 2, and 4 h time points, culture media were collected, and shed syndecan-1 in the supernatant was evaluated by slot blot analysis. The bands represent the shed syndecan-1 in the supernatant. Results are representative of two independent experiments. Clear accelerated syndecan-1 shedding in the PMA, thrombin, and EGF-treated cells relative to DMSO-treated cells was observed, and maximum shedding was observed at 4 h post treatment (Figure 4A). Densitometry quantification of the total syndecan-1 shed post treatment was cumulatively calculated for all the four time points (0.5, 1, 2, 4 h) demonstrates that there was more than a two-fold increase in syndecan-1 shedding in PMA and thrombin-treated cells and more than a one-fold increase in EGF-treated cells in both HeLa and HCE cell lines (Figure 4B). To prove that shed syndecan-1 in the supernatant of the treated cells is due to its loss from the cell surface, flow cytometry was performed (Figure 4C). The cells were treated similarly for the slot blot, and the supernatant was collected 4h post treatment. The histogram clearly shows the reduced percentage of syndecan-1 expression on the PMA, thrombin, or EGF-treated cells relative to the DMSO (control) cells.

### 3.5. PMA, Thrombin, or EGF Treatment Reduces Syndecan-1 and Heparan Sulfate Cell Surface Expression from HeLa and HCE Cells

**Figure 5:**
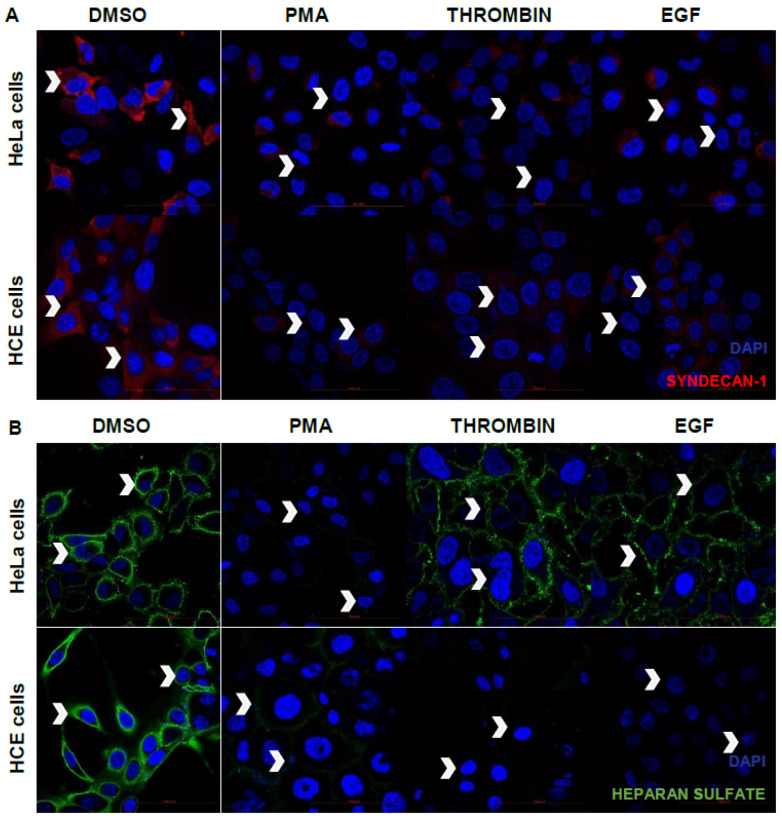
PMA, thrombin, and EGF treatment reduces syndecan-1 and heparan sulfate cell surface expression from HeLa and HCE cells. (**A**) Immunofluorescence microscopic images of the cell surface expression of syndecan-1 on HeLa cells and HCE cells after treatment with PMA (1 µM), thrombin (10 ng/mL), and EGF (5 mg/mL) in serum-free media. Nuclei are stained blue with DAPI, and syndecan-1 is stained red with anti-Alexa fluor 647 antibody. Arrows indicate the syndecan-1 shedding from the cell surface relative to the DMSO (control)-treated cells at ×63 magnification. (**B**) Immunofluorescence microscopic images of syndecan-1 on HeLa cells and HCE cells after treatment with PMA (1µM), thrombin (10 ng/mL), and EGF (5 mg/mL) in serum-free media. Nuclei are stained blue with DAPI, and heparan sulfate is stained green with FITC-conjugated antibody. Arrows indicate the clipped heparan sulfate from the cell surface at ×63 magnification.

In order to validate the observations that syndecan-1 shedding is accelerated by PMA, thrombin and EGF, immunofluorescence microscopy was performed. Upon staining the cell surface for syndecan-1, it was clearly observed that most of the syndecan-1 was intact on the cell surface, when treated with DMSO (control), whereas most of the syndecan was lost from the cell surface upon treatment with PMA, thrombin and EGF after 4 h of its application in the serum-starved media (Figure 5A). The basal cell surface expression of syndecan-1 and HS was also shown by flow cytometry studies for both HeLa and HCE cells relative to the non-stained population (Figure S3). Some studies speculate that thrombin and EGF mediate the syndecan-1 shedding by HPSE overexpression, and it was known from earlier studies that HPSE overexpression leads to heparan sulfate cleaving from the cell surface. To observe the HS expression on the cell surface post treatment, cells were treated similarly and surface stained for heparan sulfate. An accelerated heparan sulfate loss was found on HeLa and HCE cell surfaces when followed by PMA, thrombin, and EGF treatment relative to DMSO (control)-treated cells (Figure 5B).

### 3.6. Treatment with PMA, Thrombin, or EGF Enhances Virus Release

**Figure 6:**
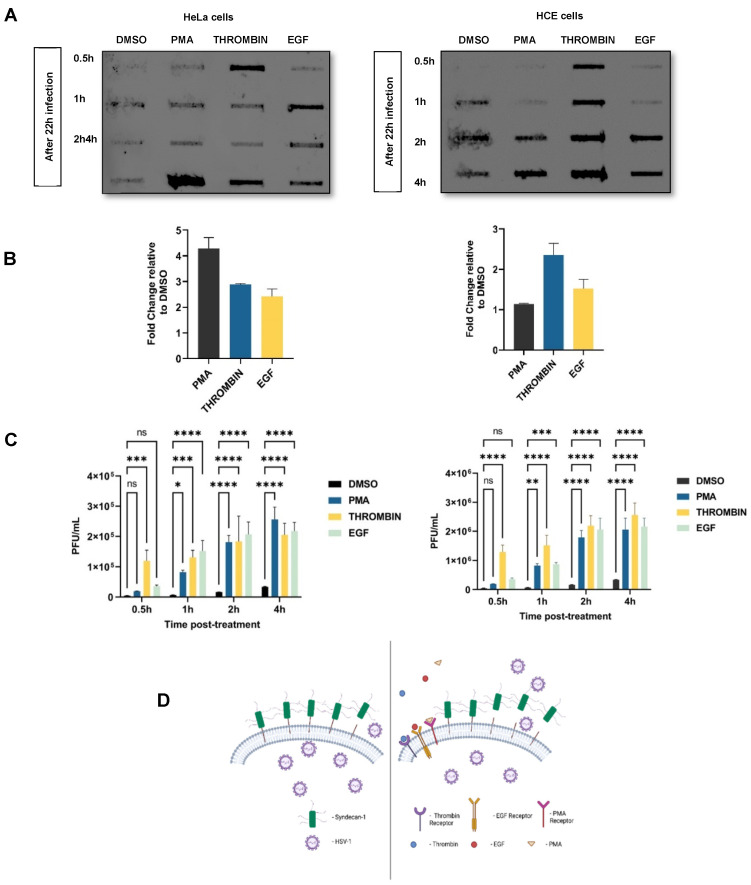
Treatment with PMA, thrombin, or EGF enhances virus release. (**A**) HeLa and HCE cells were infected with HSV-1 (KOS) for 2 h. Then, the media was replaced with complete media for 8 h. Then, the cells were washed thrice by serum-starved media and overlaid with the serum-starved media for 12 h. After a total 22 h of infection, the cells were treated with DMSO, PMA, thrombin, and EGF in serum-free media. At 0.5, 1, 2, and 4, h time points, culture media were collected. The supernatant was used to measure the gD by slot blot analysis. Results are representative of two independent experiments. Representative blots of two independent experiments are shown. (**B**) Densitometry quantification of the slot blot was performed using ImageQuant TL image analysis software, and the results are shown as fold change of gD in the supernatant in the treated cells over DMSO (control)-treated cells. Representative graph of two independent experiments conducted in duplicate is shown. (**C**) HeLa and HCE cells were infected in the similar way as done for the slot blot analysis for analyzing gD in the supernatant and treated in the same way to collect 100 µl of supernatant after each time point of 0.5, 1, 2, and 4 h. The collected supernatants were titered on a Vero cells monolayer. The Results are mean of ± 1 SD of three independent experiments (ns: non-significant; * *p* < 0.05; ** *p* < 0.01; *** *p* < 0.001; **** *p* < 0.0001). (**D**) Diagrammatic representation of the results depicting that the treatment of cells with PMA, thrombin, or EGF (shown on the left side of the diagram) accelerates the process of syndecan-1 shedding in the culture supernatant relative to the non-treated cells (shown on the right side of the diagram) and results in enhanced viral egress. Image was created using Bio Render application.

Initial results suggest an increase in active HPSE expression in HSV-1 infected cells and the involvement of HPSE in HSV-1 release from infected cells. Additionally, it has been shown that HPSE enhances the shedding of syndecan-1 from tumor cells [26]. Therefore, enhanced virus release from HSV-1 infected cells by HPSE overexpression and exogenous heparinases might be, at least partially, influenced by the induced syndecan-1 shedding by HPSE. Similarly, the same effect of PMA, thrombin, and EGF on the syndecan-1 shedding was also observed. Now, to understand the accelerated effect of syndecan-1 shedding by PMA, thrombin and EGF treatment on virus release from infected cells, the following set of experiments were performed. Both HeLa and HCE cells were infected at an MOI of 0.01 with HSV-1 (KOS) for 2 h. After 2 h, complete media was added, and incubation was continued for 8 h. Then, cells were serum starved for 12 h, which was followed by PMA treatment (1 µM), thrombin treatment (5 mg/mL), and EGF treatment (10 ng/mL) in serum-free media for 0.5, 1, 2, and 4 h, and the supernatant was collected at each time point. Control cells were DMSO treated. The effect of PMA, thrombin, and EGF on viral egress by syndecan-1 shedding was observed by analyzing the gD of the egressed virus post treatment through a slot blot analysis (Figure 6A). The bands here represent the gD from the egressed virus that was also found to be maximum at 4 h post treatment, which was similar to maximum syndecan-1 shedding at the same time point earlier. The densitometry analysis of the total gD measured in the culture supernatant in 4 h treatment of the cells with PMA, thrombin and EGF showed total enhanced viral egress after treatment relative to the DMSO (control)-treated cells (Figure 6B). To quantify the total infectious particles released post treatment, plaque assay was performed after 0.5, 1, 2, and 4 h time points. HSV-1 released to the culture supernatant was evaluated by titering 100 µL of culture supernatant on Vero cells. PMA, thrombin, and EGF treatment resulted in an enhanced HSV-1 release from infected cells to the culture media, with the most significant increase to be at later time points (2 and 4 h) (Figure 6C). Figure 6D shows the diagrammatic representation of the results depicting that the treatment of cells with PMA, thrombin and EGF accelerates the process of syndecan-1 shedding in the culture supernatant, resulting in enhanced viral egress.

## 4. Discussion

HPSE modulates HSPGs in multiple ways. Not only does HPSE have the function of degrading HS chains, but also it enhances the shedding of HSPGs especially syndecan-1 from the cell surface. HSPGs, which are the main attachment receptors for HSV-1, contribute to HSV-1 infection both at the level of HS chains and at the level of the protein core particularly at the step of virus attachment and cell-to-cell spread. The effect of HPSE modulation on HSPGs upon HSV-1 infection has always been an area of interest for our research. This study highlights the role of HPSE in HSV-1 release from infected cells by syndecan-1 shedding. HPSE overexpression and exogenous heparinases treatment significantly induced virus release from infected cells to the supernatant. While HSPGs are well known for their important contribution during early events of viral infection, our results show that the same receptors show hindrance during its egress, which is a later step of the lytic phase of the infection. HPSE’s role in HSV-1 egress provides an important spread mechanism for the virus. Viral progeny benefit from leaving an already infected and probably dying cell to reach a healthy cell to start a new round of infection. Importantly, since HSPGs serve as attachment receptors for many viruses, including hepatitis C virus (HCV), human papillomavirus, and Dengue virus, it is tempting to speculate that HPSE might also be involved in the egress of these viruses [34,35,36]. Although HSPGs provide attachment receptors for HSV-1, HPSE overexpression did not have an effect on HSV-1 entry into the host cell and virus-induced cell-to-cell fusion. One possible explanation is that the induced HPSE is insensitive to HS modifications that are important for virus entry. Another possibility is that inducing the expression of HPSE resulted in an enzyme that was inactive when HSV-1 entry and cell fusion were assessed; thus, it was unable to degrade HS chains. Interestingly, HPSE overexpression resulted in fewer plaques inside the cells as compared to control cells overexpressing an empty vector. Since HPSE overexpression has no effect on virus entry but yet induces virus egress significantly, it is plausible that the induced virus release from infected cells disrupted the efficiency of virus plaque formation. As the plaque assays were performed with methylcellulose overlaying the cells, the plaque formation is dependent on virus lateral cell-to-cell spread. In this case, cells with enhanced HPSE expression have fewer virus available on the cell surface for lateral cell-to-cell spread because more virus is released to the cell culture supernatant and trapped in the methylcellulose mush. Plaquing efficiency also depends on the efficiency of virus replication. However, since enhanced HPSE expression in infected cells showed significantly higher virus titer in cell culture supernatant compared to the titer of the control cells’ supernatant, virus replication could also be affected by inducing HPSE expression. HPSE exerts enzyme-dependent and enzyme-independent functions. The active form of HPSE accounts for the HS degrading enzyme-dependent functions. Since our early findings have shown that HSV-1 infection induced the expression of active HPSE, which correlated with a decrease in the inactive form of the enzyme, this suggests that HSV-1 infection induces HS degradation [37,38]. This might be exploited by the virus to facilitate HSV-1 release and thus subsequent spread to uninfected cells, and possibly transmission to a new host. This effect on HPSE expression was also observed in HCV infection, where HPSE expression was elevated in HCV-related hepatocellular carcinoma (HCC) compared to HCV-negative HCC [28,30]. This observation is important in the sense that not only do HSPGs on the cell surface contribute to HSV-1 infection, but HSV-1 modulates HSPGs to support the infection process. Since HPSE enhances the shedding of syndecans from the cell surface, accordingly, we found that enhancing syndecan-1 shedding by treating infected cells with the shedding agonist PMA, thrombin, and EGF resulted in an increase in virus release to the cell culture supernatant. This suggests that HPSE enhances virus release not only through degrading HS chains but also through increasing syndecans shedding from the surface of the infected cell. In a previous study, PMA and forskolin treatment has been found to trigger active HPSE secretion [31]. PMA is a strong Protein Kinase C (PKC) inducer, while forskolin is a Protein Kinase A (PKA) inducer. Utilizing PKC and PKA inhibitors, it has been shown that active HPSE secretion is accelerated by extracellular cues activating PKA and C signaling pathways [31]. Therefore, the observed induction of HSV-1 release after PMA treatment may also be a result of enhanced active HPSE secretion. Interestingly, PKC activation has been reported in HSV-1 infection at 8 h and 12 h post infection [32]. HSV-1 activates and recruits PKC to the inner nuclear membrane to phosphorylate lamin B to help modify the nuclear lamina and mediate the budding of nucleocapsids at the inner nuclear membrane during virus egress [32]. Therefore, it is possible that HSV-1-mediated PKC activation facilitates further release of the viral particles at the cellular plasma membrane by inducing active HPSE secretion. HPSE has been shown to be an important player in tumor progression and metastasis. HPSE expression has been shown to be elevated in virtually all human carcinomas examined [24]. This is attributed to enhanced cell dissemination as a result of HS cleavage and remodeling of the ECM and basement membrane underlying epithelial and endothelial cells, which act as a barrier to tumor cell invasion and spread [24]. Furthermore, HPSE mediates the establishment of a vascular network that promotes primary tumor growth and metastatic cells invasion [24]. It is fascinating to anticipate that HSV-1 exploits HPSE disseminating functions to induce its spread the same way tumor cells utilize HPSE functions to induce their invasion and metastasis. Additionally, the compounds PMA, thrombin, and EGF, which we have shown as accelerators of syndecan-1 shedding in the HeLa and HCE cell lines, contribute to an enhanced viral egress. These compounds activate signaling pathways that lead to an increase in syndecan-1 ectodomains in the supernatant, although the exact pathway is not completely understood. As syndecan-1 is the main source of heparan sulfate, the removal of the extracellular components of syndecan-1 would lead to the removal of the hindrances to viral egress. As HPSE is implicated in the accelerated shedding of syndecan-1, it is a possibility that the PMA, thrombin, and EGF pathways may lead to an increase in HPSE expression.

A limitation of our study is that transient overexpression of HPSE was used in some experiments. In the future, the use of cell lines stably expressing HPSE may provide a more useful resource for studying the regulators of HPSE shedding and how they may be affecting HSV release. A possible caveat is that cells overexpressing constitutively active HPSE, given its emerging pro-viral/pro-microbial roles, may be more prone to death due to high susceptibility to infection combined with the inflammatory conditions that its constitutive expression may generate. Additional studies are needed to better understand the role and regulation of syndecan-1 shedding and how cytokine and growth factor activity might impact this process. While several HPSE inhibitors are currently under clinical trials as novel anti-cancer therapeutics, it is tempting to study the effect of these inhibitors on HSV-1 infection in an in vivo model of infection.

## Data Availability

Data supporting the findings of this study are available within the article [and/or] its Supplementary Materials.

## Supplementary Materials

The following are available online at https://www.mdpi.com/article/10.3390/v13091748/s1, Figure S1. Microscopic images of pIRES2-EGFP-HPSE transfected cells at 48 h post-transfection, Figure S2. MTT ASSAY to represent % cell viability of PMA, thrombin and EGF in HeLa and HCE cells, Figure S3. Cellular level expression of A. syndecan-1 and B. heparan sulfate (HS) for HeLa and HCE cells.
