# Supplementary material for: Protease, Growth Factor, and Heparanase-Mediated Syndecan-1 Shedding Leads to Enhanced HSV-1 Egress
Source: Viruses. 2021 Sep 1;13(9):1748. doi: 10.3390/v13091748 (PMC8473078; doi:10.3390/v13091748)
Supplement: Supplementary file 1 [file viruses-13-01748-s001.zip › viruses-1313062-supplementary.pdf]

SUPPLEMENTARY DATA

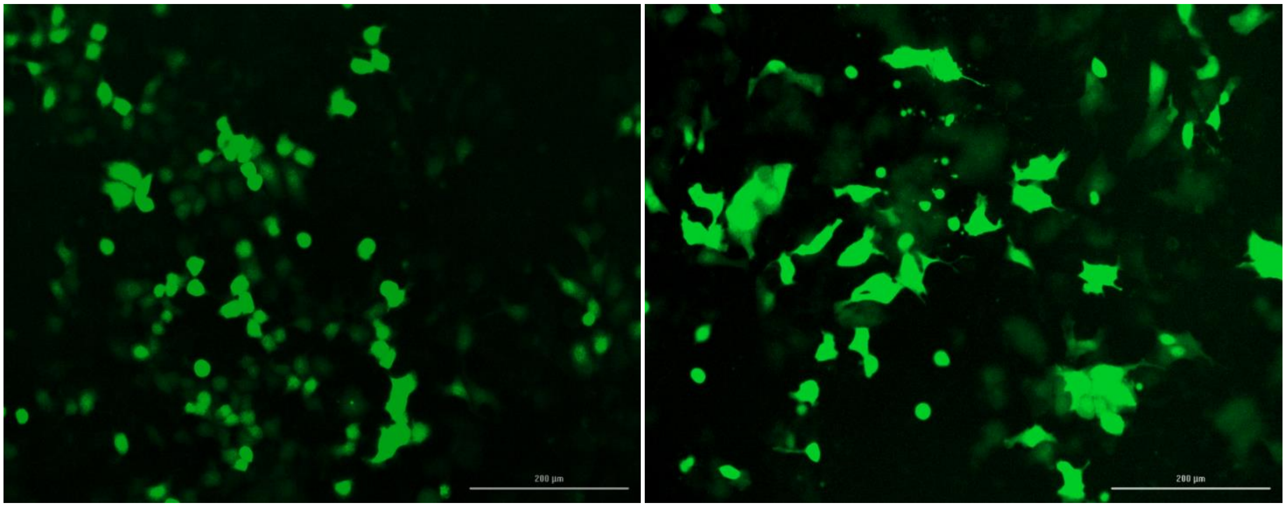

HeLa cells **HCE cells**

Fig S1. Microscopic images of pIRES2-EGFP-HPSE transfected cells at 48h post-transfection.

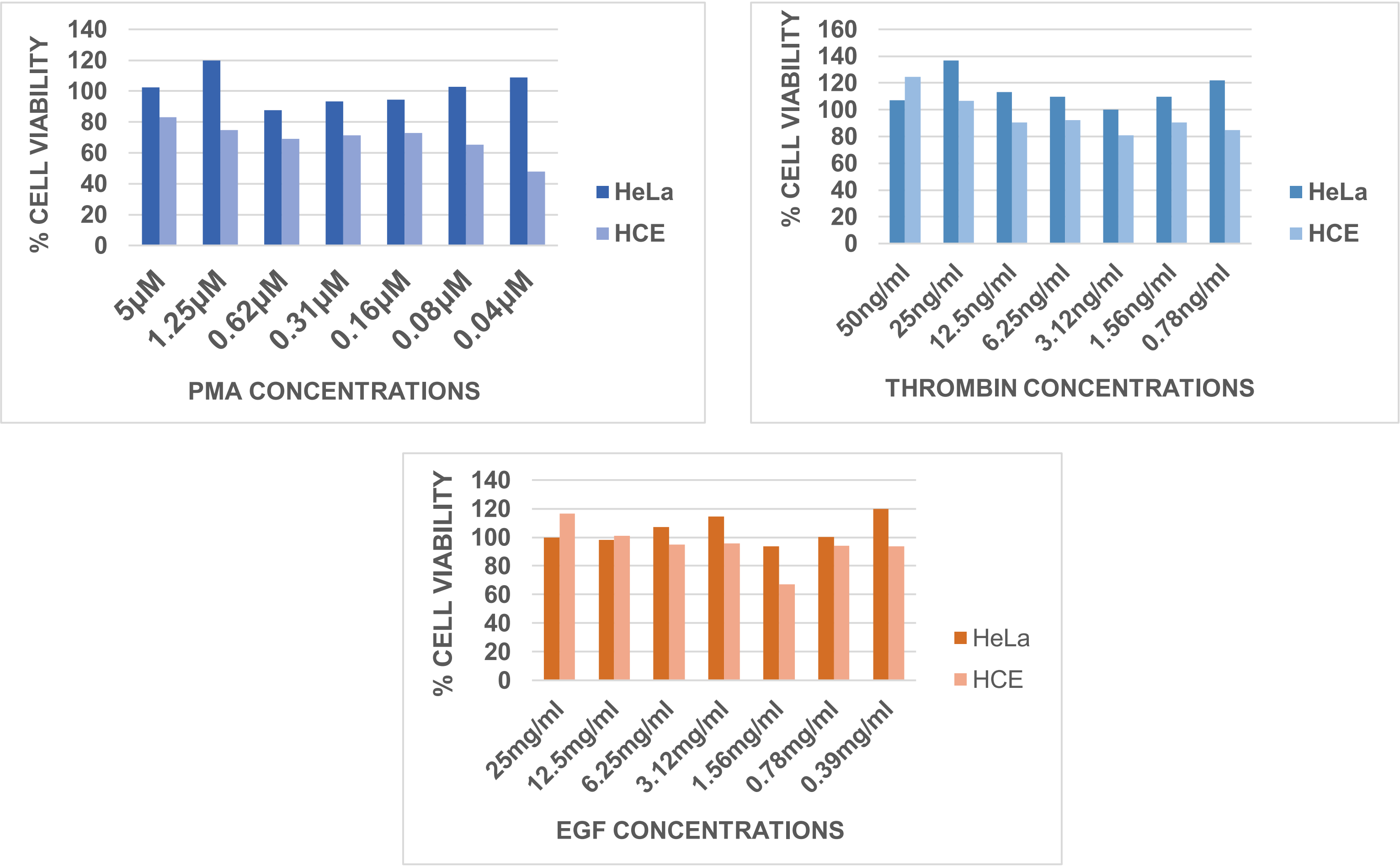

Fig S2. MTT ASSAY to represent % cell viability of PMA, thrombin and EGF in HeLa and HCE cells.

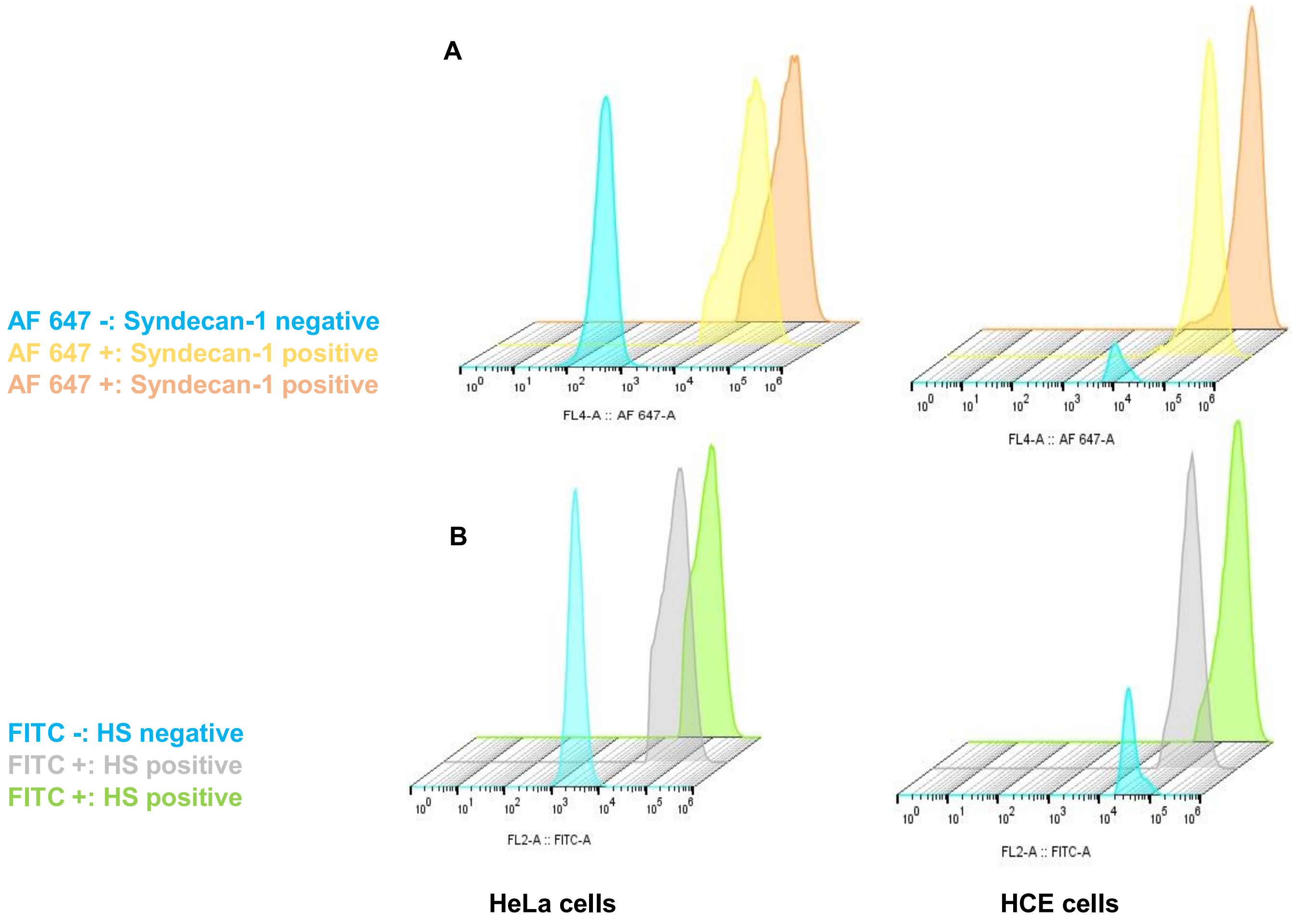

Figure S3: Cellular level expression of A. syndecan-1 and B. heparan sulfate (HS) for HeLa and HCE cells.
